# Human third-party observers accurately track fighting skill and vigour along their unique paths to victory

**DOI:** 10.1038/s41598-022-19044-4

**Published:** 2022-09-01

**Authors:** Neil R. Caton, Barnaby J. W. Dixson

**Affiliations:** 1 School of Psychology, University of Queensland, Brisbane, QLD Australia; 2 School of Health and Behavioural Sciences, University of the Sunshine Coast, Sunshine Coast, QLD Australia; 3 Division of Psychology, University of Stirling, Stirling, Scotland, UK

**Keywords:** Sexual selection, Psychology

## Abstract

Sexual selection via male-male contest competition has shaped the evolution of agonistic displays, weaponry, and fighting styles, and is further argued to have shaped human psychological mechanisms to detect, process, and respond appropriately to cues of fighting ability. Drawing on the largest fight-specific dataset to date across the sports and biological sciences (*N* = 2765 fights), we examined how different indicators of fighting ability in humans reflect unique paths to victory and indicate different forms of perceived and actual resource-holding power (RHP). Overall, we discovered that: (1) both striking skill and vigour, and grappling skill and vigour, individually and collectively predict RHP; (2) different RHP indicators are distinguished by a unique path to victory (e.g., striking skill is a knockout-typical strategy, whereas grappling vigour is a submission-typical strategy); and (3) third-party observers accurately track fighting skill and vigour along their unique paths to victory. Our argument that different measures of RHP are associated with unique paths to victory, and third-party observers accurately track fighting vigour and skill along their unique paths to victory, advance our understanding not only of human contest competition, but animal contest theory more broadly.

## Introduction

Sexual selection via male-male contest competition has shaped the evolution of agonistic displays, fighting styles, and weaponry^1,2^. During contests, horns, tusks, antlers and claws are employed to directly inflict harm on conspecifics^2,3^, while ornamental features broadcast social rank and dominance^4,5^. Both mechanisms are tied to male resource holding potential (RHP), wherein individuals compete for favourable territories and social rank to secure reproductive opportunities^6,7^. Fighting ability and social dominance may have evolved as honest signals of RHP, so that only individuals with greater underlying biological quality can bear the costs of outwardly displaying formidability to conspecifics^8,9^. Despite the fact that Darwin^10^ argued that humans evolved for “striking or fighting with an enemy” a century and a half ago, researchers have only recently turned their attention to fighting skill and vigour in the context of human contest competition^11^.

Hand-to-hand combat is an ancient feature of human contest competition^12,13^, potentially driving adaptations that increase fighting ability and RHP. Striking an opponent with one’s fists during an agonistic exchange is universal among Europeans^10,13^, is a central component of Asian martial arts (e.g., Karate; Kung Fu; the Chinese martial art Xing Yi Quan means “form and intent fist”), appears in humanity’s earliest written sources (e.g., Exodus 21:18; Ezekiel 6:11; Matthew 26:67), occurs among the Yanomamö of Brazil hunter-gatherers in Brazil and Venezuela^14^ and accounts for 46–67% of fight-associated facial fractures in contemporary populations^12^. There is an emerging body of evidence that supports the argument that hand-to-hand combat has been a powerful selective pressure throughout our species’ evolutionary history, shaping men’s fist morphology^13^, arm power and force^15^, neck musculature^16^, and arm length and shoulder breadth^17^.

In addition to driving the evolution of morphological structures employed in contest competition, sexual selection is argued to have shaped human psychological mechanisms to detect, process, and respond appropriately to cues of fighting ability^18,19^. People show strong agreement across cultures which male faces look socially dominant and aggressive^20^ and using faces alone can accurately predict men’s physical strength^21^ and the winners of fights^22,23^. Moreover, men displaying clear physical indicators of RHP—including overall body size, total upper body strength, shoulder breadth, bicep size, handgrip strength, and neck musculature—are perceived as stronger^16,24^, more attractive^16,25,26^, more aggressive^22,27^, better fighters^16,22^, are more likely to be elected as leaders in politics and business^28,29^, and have higher mating and reproductive success^30–32^.

Sexual selection is also proposed to have favoured behavioral processes to deploy those fighting strategies and techniques that maximise the potential to inflict injury on an opponent^33^. These include fighting vigour, defined as the rate at which fighting manoeuvres are performed, and fighting skill, which refers to the capacity to effectively enact aggressive manoeuvres during agonistic exchanges^33^. A review of the contest competition literature in nonhuman animals indicates that a large swathe of research implies that fighting skill contributes to RHP (e.g., Mantis shrimp, *Neogonodactylus bredini*; Richardson's ground squirrels, *Spermophilus richardsonii*)^34^, with recent evidence directly showing that fighting skill contributes to RHP in hermit crabs^35,36^. The structural weapons that are used during fights may have been maintained by the emergence of specific fighting styles^3,37^, furthering the argument that sexual selection operates on behavioural processes that deploy fighting strategies and techniques during contest competition, such as fighting skill and vigour.

Although similar tests of contest competition in humans are sparse, Lane and Briffa^11^ reported that striking vigour contributed to RHP in mixed-martial-arts (MMA) fights (*N* = 548) from the Ultimate Fighting Championship© (UFC). An interaction between striking vigour and striking skill was also reported, such that the effect of vigour on the likelihood of victory increased with skill, while there was no overall effect of striking skill on the likelihood of victory independent of vigour^11^.

Despite a long tradition of wrestling across cultures^38^ that is documented in ancient written sources (e.g., Genesis 32:22–31; Hosea 12:3–5) and observed in hunter-gatherer tribes^38^, whether grappling skill and vigour are indicators of RHP is still unknown^11^. Grappling involves gripping and grabbing an opponent, either leading to or during ground control, eventuating in the conspecific conceding defeat (termed submission). Attempts to submit an opponent are indeed defined as attempts to end the contest via grappling, locks, and chokes^39^, suggesting that grappling manoeuvres are an important contributor of submission victories. Indeed, submissions are sometimes specifically referred to as grappling submissions^40^, indicating the inextricable link between grappling abilities and submission victories. Recent evidence suggests that humans evolved morphological features that signal their grappling abilities, as all major facial width-to-height ratio measurements—automatic and manual measurements, using both eyelid and eyebrow landmarks—predicted men’s grappling behaviours in 1367 UFC fights, before and after controlling for 17 different control variables^23^.

Furthermore, whether fighting vigour and skill predict KO/TKO-typical, submission-typical strategies, or all-round indicators of RHP remains to be determined. For example, striking skill may be more strongly associated with KO/TKO victories whereas grappling vigour may be more strongly associated with submission victories. Evolutionarily, only one accurate but powerful strike would have been necessary to render an opponent unconscious^41,42^ whereas the process leading to a successful submission can be arduous, requiring continual efforts to grapple and lock the opponent^40^. This proposition that humans use alternative fighting tactics to their advantage is congruent with non-human animal research, in which animals (e.g., green anole lizard, *Anolis carolinensis*) adopt distinct fighting styles based on their morphology (e.g., greater jumping velocity and acceleration in lightweight males but greater biting force in heavyweight males)^43^.

Animal psychological systems have evolved to detect, process, and respond to agonistic behaviours deployed during contest competition^1,10,19^. This is the case for both non-human animals directly involved in the contest^1,10,19^ and third-party observers of these contests^44–46^. Given that human evolutionary history has been characterised by both group-based living and contest competition^12,14,18,19^, human third-party observers should possess psychological systems that allow them to gauge conspecifics’ fighting skill and vigour^11^. More specifically, if human fighting abilities are associated with unique paths to victory, then we should expect that third party judges’ evaluations should accurately track the specific fighting manoeuvres’ typical avenue to victory, or “be analogous to the decision the loser would eventually make for themselves if the fight were to continue”^11^.

### The present study

The present study uses the most comprehensive data set to date (*N* = 2765 fights) to test how different indicators of human fighting ability reflect unique paths to victory and hence different forms of RHP. Moreover, we sought to test whether third-party observers accurately track these predictors of RHP along their unique paths to victory. First, we hypothesised that striking skill, vigour, as well as the interaction between striking vigour and skill would increase the likelihood of victory (Hypothesis 1). Next, we hypothesised that grappling skill, vigour, as well as the interaction between grappling vigour and skill would increase the likelihood of victory (Hypothesis 2).

Striking skill should share stronger associations with KO/TKO than submission victories, whereas striking vigour should be evenly associated with victories from KO/TKO and submission (Hypothesis 3). We postulated that striking vigour would be an all-round strategy to victory because conspecifics must repeatedly strike their opponent to emerge victorious by either knockout or submission. Fighters are required to repeatedly strike their opponent to render them incapacitated or unconscious, and similarly, submissions require vigorously striking an opponent to keep them in place—to maintain the opponent’s submissive position^40^.

We argued that striking skill acts as a KO/TKO-typical path to victory because, evolutionarily, only one accurate but powerful strike was necessary to render an opponent unconscious^41,42^. Striking skill would not predominate as a submission-typical strategy because most strikes in a submission-oriented position (ground or clinch) would successfully land, as combatants are within each other’s immediate proximity^23^. There would have therefore been minimal variance in striking skill during submission attempts throughout our species’ evolutionary history of contest competition, and thereby reduced selective pressures on striking skill as a submission-typical strategy.

With respect to grappling techniques, we postulated that grappling skill would be equally associated with both KO/TKO and submission victories and that grappling vigour would be more strongly associated with submission than with KO/TKO victories (Hypothesis 4). We postulated that grappling skill would act as an all-round strategy to victory because only one skilfully enacted takedown can lead to a successful *ground and pound* where, after the opponent has been taken to the ground, a series of strikes are repeatedly landed on the opponent^23^. This places the opponent in a highly susceptible position, in which they can quickly be rendered unconscious or submitted^23^. We postulated that grappling vigour would be a submission-typical indicator of RHP because, unlike when *one* skilfully enacted takedown can lead to a knockout, *continual* takedown attempts in a short period of time could serve as an indicator of the individual’s intent to win by submission because submission victories require the continual and arduous enactment of grappling manoeuvres in ground combat to outmanoeuvre the opponent^40^.

If third-party observers accurately track striking skill as a KO/TKO-typical indicator of RHP, then striking skill should be more important for bystander evaluations than submissions for victory but equally important to bystander evaluations as KO/TKOs for victory. Likewise, if third-party observers accurately track striking vigour as an all-round indicator of RHP, then striking vigour should be equally important for bystander evaluations as it is to KO/TKO and submission victories (Hypothesis 5). If third-party observers accurately track grappling skill as an all-round indicator of RHP, then grappling skill should be equally important for bystander evaluations as it is to KO/TKO and submission victories. If third-party observers accurately track grappling vigour as a submission-typical indicator of RHP, then grappling vigour should be more important for bystander evaluations than KO/TKOs for victory but equally important to bystander evaluations as submissions for victory (Hypothesis 6).

## Results

### **Hypothesis 1. Striking skill and vigour increase the likelihood of victory.**

There was a significant association between striking skill and the likelihood of victory (*β* = 1.19 ± 0.10, *X*^2^ = 12.43, *p* < 0.001), such that striking skill increased the likelihood of victory. There was a significant association between striking vigour and the likelihood of victory (*β* = 1.03 ± 0.10, *X*^2^ = 10.30, *p* < 0.001), such that striking vigour increased the likelihood of victory. There was also a significant positive interaction between striking skill and striking vigour on the likelihood of victory (*β* = 0.35 ± 0.07, *X*^2^ = 4.91, *p* < 0.001), which indicates that the probability of winning increased with striking skill and this effect of skill was enhanced by striking vigour. There were no significant effects for sex or its interactions.

### **Hypothesis 2. Grappling skill and vigour increase the likelihood of victory.**

There was a significant association between grappling skill and the likelihood of victory (*β* = 0.72 ± 0.07, *X*^2^ = 10.66, *p* < 0.001), such that grappling skill increased the likelihood of victory. There was a significant association between grappling vigour and the likelihood of victory (*β* = 0.24 ± 0.08, *X*^2^ = 3.02, *p* = 0.003), such that grappling vigour increased the likelihood of victory. There was a significant positive interaction between grappling vigour and skill (*β* = 0.31 ± 0.07, *X*^2^ = 4.65, *p* < 0.001), such that the probability of winning increased with grappling skill and was enhanced by grappling vigour. There were no significant effects for sex or its interactions.

### **Hypothesis 3. Striking skill is a KO/TKO rather than submission strategy, whereas striking vigour is evenly associated with KO/TKO and submission.**

**Figure 1:**
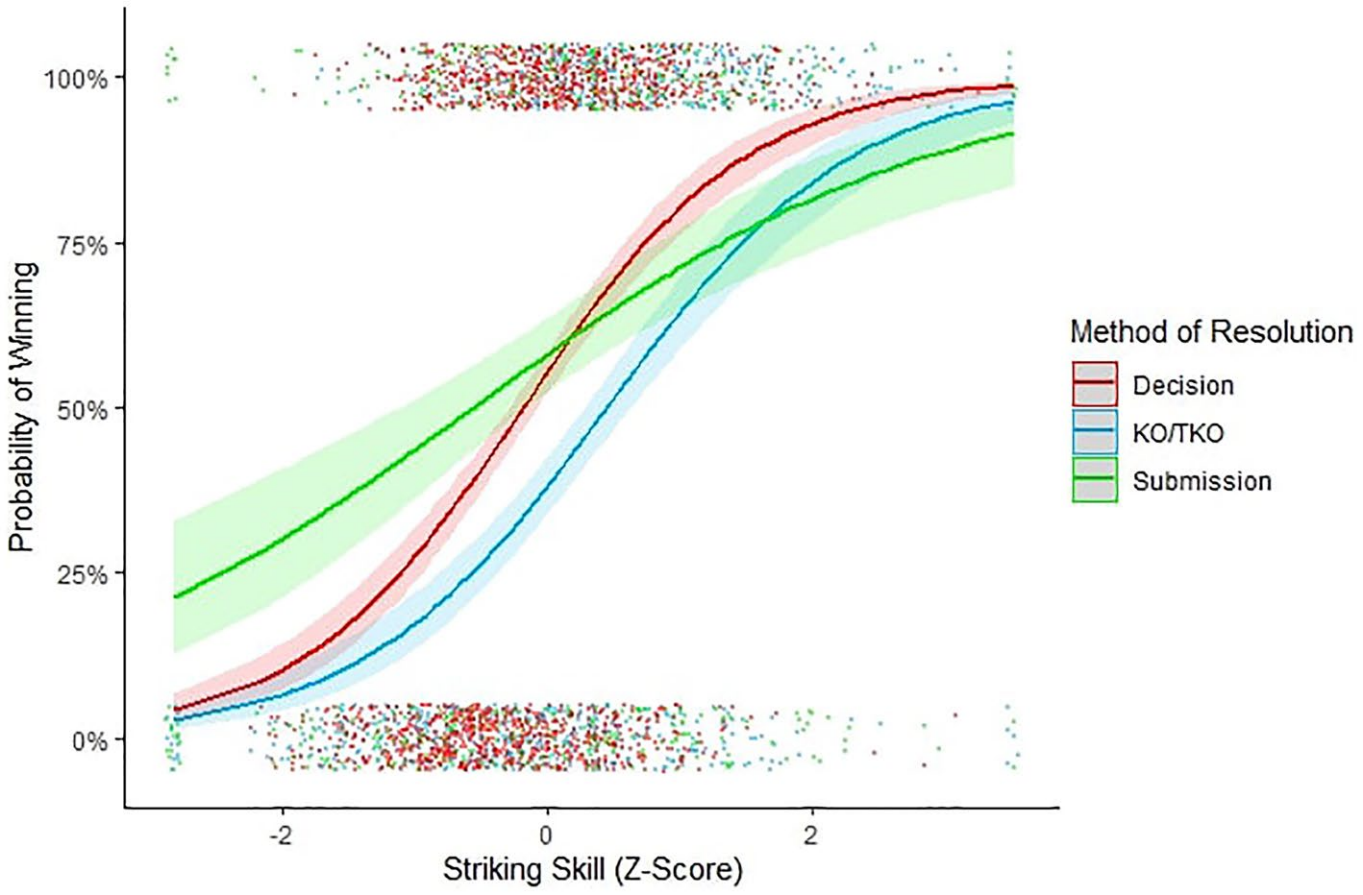
Interaction between striking skill and method of resolution on likelihood of winning. Error bands illustrate 95% confidence intervals. Data points are jittered for visibility purposes.

**Figure 2:**
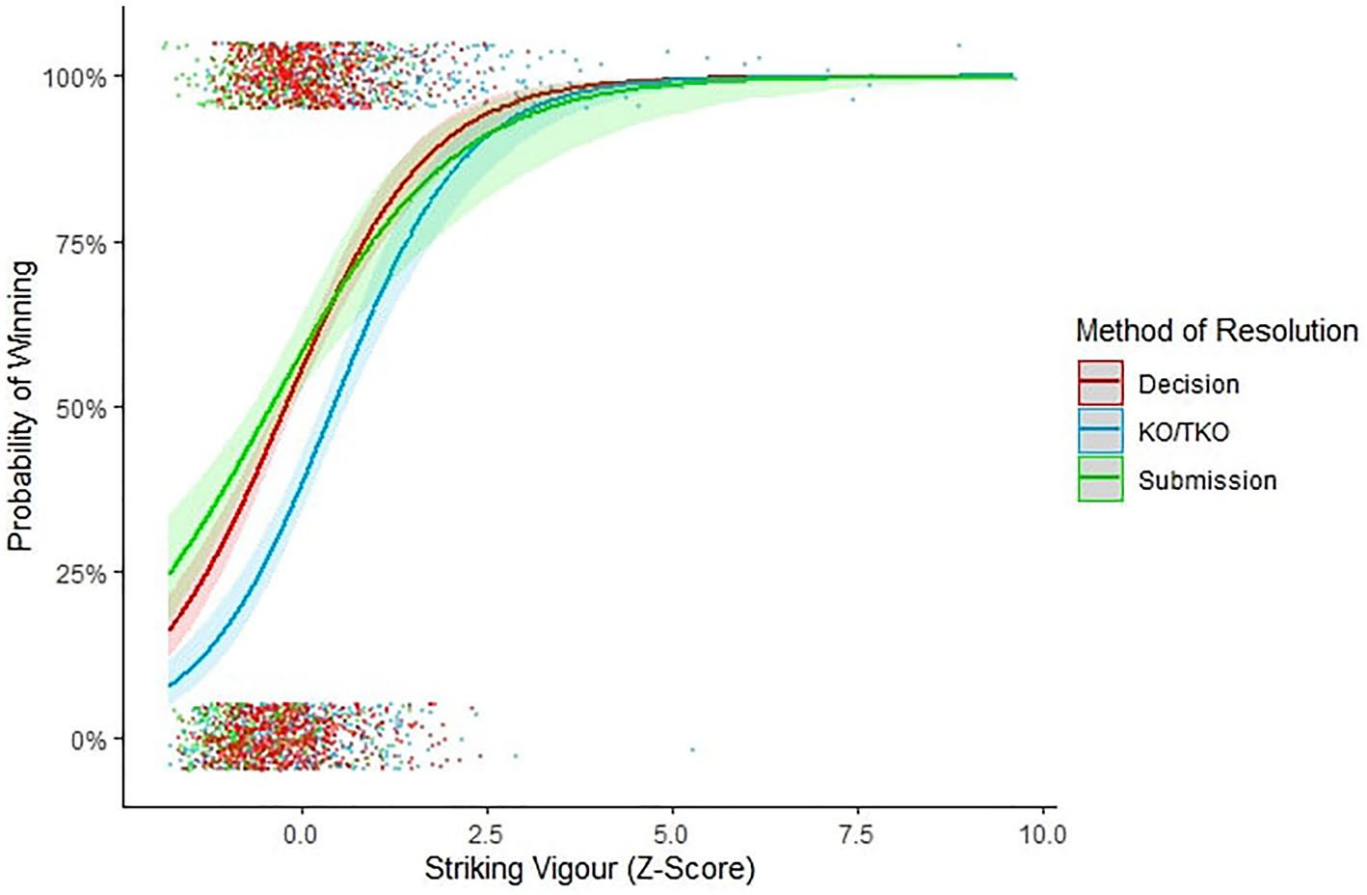
Interaction between striking vigour and method of resolution on likelihood of winning. Error bands illustrate 95% confidence intervals. Data points are jittered for visibility purposes.

There was a stronger association between striking skill and KO/TKO victories than with submission victories (*β* = − 0.48 ± 0.14, *X*^2^ = − 3.54, *p* < 0.001) indicating that striking skill was a KO/TKO-typical strategy (Fig. 1). Striking vigour was equally associated with both KO/TKO and submission victories (*β* = − 0.31 ± 0.18, *X*^2^ = − 1.74, *p* = 0.08) indicating that striking vigour was an all-round strategy to victory (Fig. 2).

### **Hypothesis 4. Grappling skill is equally associated with KO/TKO and submission victories, while grappling vigour is associated with submission and not KO/TKO victories.**

**Figure 3:**
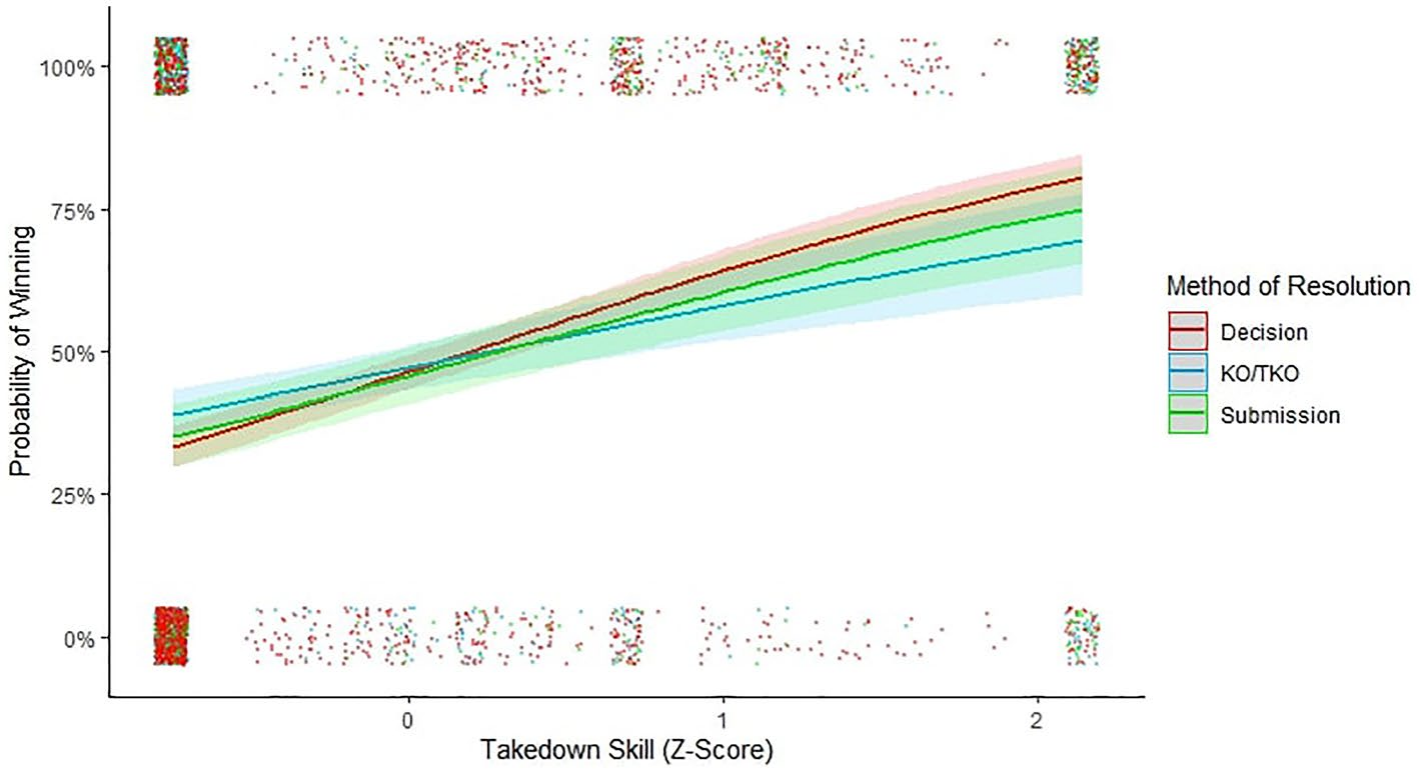
The interaction between grappling skill and method of resolution on the likelihood of winning (decision, KO/KTO, and submission). Error bands illustrate 95% confidence intervals. Data points are jittered for visibility purposes.

**Figure 4:**
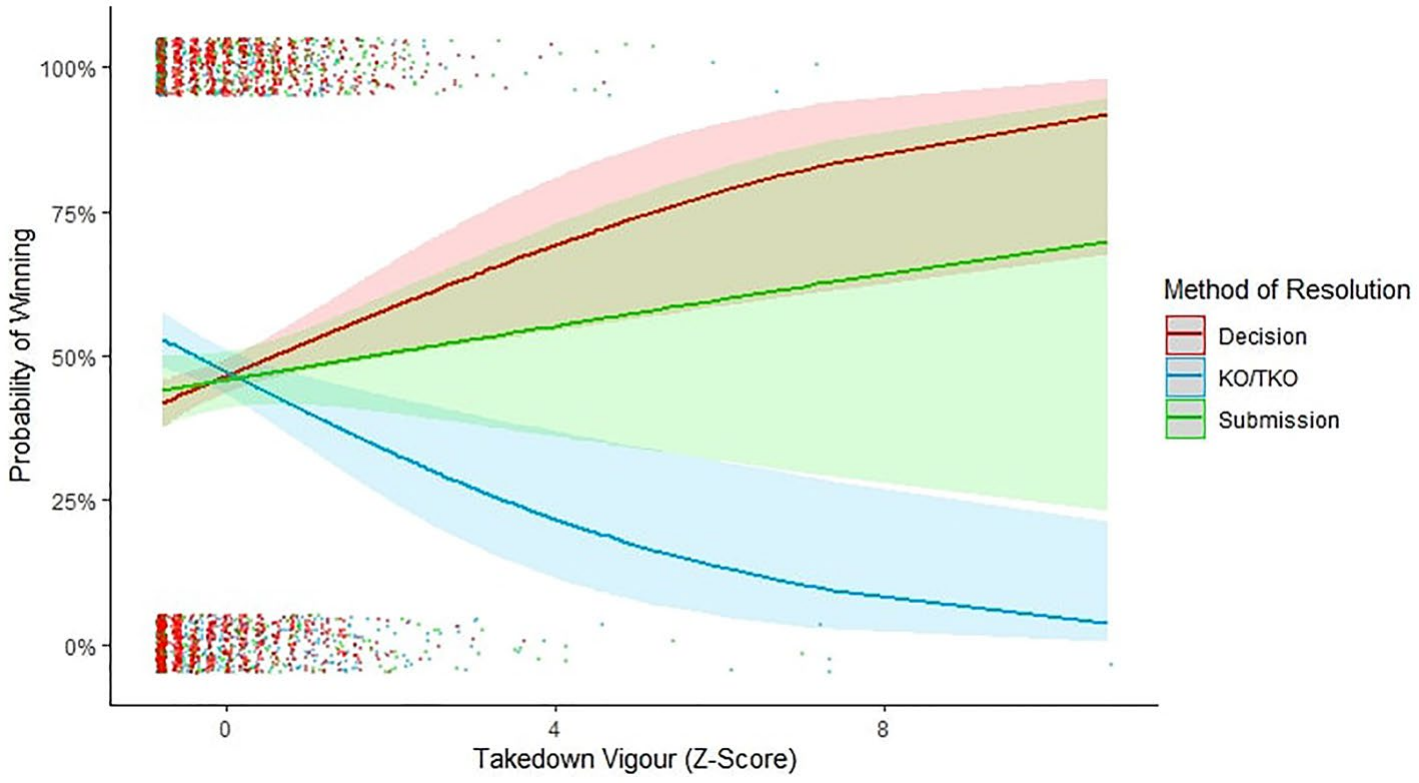
The interaction between grappling vigour and the method of resolution on likelihood of winning. Error bands illustrate 95% confidence intervals. Data points are jittered for visibility purposes.

In line with Hypothesis 4, grappling skill was not more strongly associated with either KO/TKO or submission victories (*β* = 0.13 ± 0.13, *X*^2^ = 1.01, *p* = 0.31) indicating that grappling skill was an all-round strategy to victory (Fig. 3). Grappling vigour was more strongly associated with submission than KO/TKO victories (*β* = 0.32 ± 0.13, *X*^2^ = 2.38, *p* = 0.02) indicating that grappling vigour was a submission-typical strategy (Fig. 4).

### **Hypothesis 5. Third-party observers accurately track striking skill as a KO/TKO-typical indicator of RHP; striking vigour is tracked as an all-round indicator of RHP.**

Striking skill was more important for bystander evaluations than for submissions for victory (*β* = − 0.61 ± 0.13, *X*^2^ = − 4.66, *p* < 0.001) but was equally important (i.e., analogous) for bystander evaluations as KO/TKOs for victory (*β* = − 0.11 ± 0.13, *X*^2^ = − 0.82, *p* = 0.41). Collectively, our results indicate that: (1) striking skill is, most broadly, an indicator of RHP; (2) striking skill is, more specifically, a KO/TKO-typical indicator of RHP; and (3) third-party observers (judges’ decisions) accurately tracked striking skill as a KO/TKO-typical, but not submission-typical, strategy (Fig. 1).

Striking vigour was also equally important to KO/TKO (*β* = 0.08 ± 0.15, *X*^2^ = 0.56, *p* = 0.58) and submission victories (*β* = − 0.23 ± 0.17, *X*^2^ = − 1.38, *p* = 0.17) as it was to judges’ decisions. Collectively, our results indicate that: (1) striking vigour is an indicator of RHP; (2) striking vigour is all-round indicator of RHP; and (3) third-party observers (judges’ decisions) accurately tracked striking vigour as an all-round indicator of RHP (Fig. 2).

### **Hypothesis 6. Bystanders evaluate grappling skill equally for KO/TKO and submission victories, while grappling vigour is more important for submission than KO/TKOs.**

Grappling skill was more important for bystander evaluations than for KO/TKO for victory (*β* = − 0.29 ± 0.11, *X*^2^ = − 2.67, *p* = 0.01) but was equally important (i.e., analogous) for bystander evaluations as submissions for victory (*β* = − 0.13 ± 0.12, *X*^2^ = − 1.11, *p* = 0.27) indicating that third-party observers inaccurately perceived grappling skill as a submission-typical strategy. These results indicate that grappling skill is an all-round indicator of RHP that is not distinguished by a unique path to victory, and that third-party observers inaccurately tracked grappling skill as a submission-typical indicator of RHP (Fig. 3).

Grappling vigour was more important for bystander evaluations than for KO/TKO for victory (*β* = − 0.53 ± 0.12, *X*^2^ = − 4.39, *p* < 0.001) but was equally important for bystander evaluations as submissions for victory (*β* = − 0.14 ± 0.13, *X*^2^ = − 1.15, *p* = 0.25). Combined, these results indicate that: (1) grappling vigour is an indicator of RHP; (2) grappling vigour is a submission-typical indicator of RHP; and (3) third-party observers (judges’ decisions) accurately tracked grappling vigour as a submission-typical indicator of RHP (Fig. 4).

## Discussion

Drawing on data from 2765 fights—the largest fight-specific sample to date in contest competition research—the present study supports that: (1) fighting skill in humans is an indicator of RHP that exists independently of vigour; (2) different RHP indicators are distinguished by a unique path to victory; and (3) third-party observers accurately track fighting vigour and skill along their unique paths to victory.

Striking skill, vigour, as well as their interaction, increased the likelihood of victory. This finding lends support to prior research, which showed that striking vigour and the interaction between striking vigour and skill increase the likelihood of victory in non-human^33–36^ and human^11^ contests. In the present work, we provided the first evidence that striking skill contributes to RHP independently of striking vigour. We also found that grappling skill and vigour act individually, and in concert, to predict individual differences in the pathway to victory. Performing distinct striking and grappling manoeuvres requires complex neurocognitive capacities, which suggests that fighters differ in their ability to adjust their fighting skill and efforts against their opponent. Indeed, striking skill was a KO/TKO-typical strategy whereas striking vigour was an all-round strategy to victory. Further, grappling skill was an all-round strategy to victory whereas grappling vigour was a submission-typical strategy. Our findings provide the first evidence that fighters pursue specific techniques and expend greater effort to follow specific fighting strategies along their unique paths to victory^33^. This is consistent with non-human animal research, in which animals (e.g., green anole lizard, *Anolis carolinensis*) adopt distinct fighting styles based on their individual characteristics (e.g., greater jumping velocity and acceleration in lightweight males but greater biting force in heavyweight males) ^43^.

Given that non-human animal research indicates that bystanders possess cognitive capacities to detect cues of RHP during agonistic exchanges^44–46^, we also expected that human third party spectators (i.e., UFC judges) would accurately assess individual performances within contests^11^. We report that judges’ decisions accurately tracked the association between striking skill and victories from knockouts, but not victories from submission. Judges’ decisions also accurately tracked the association between striking vigour and victories via KO/TKO and submission. Judges’ decisions further accurately tracked the positive association between grappling vigour and submission but not knockout victories, again supporting the overarching claim that judges’ rulings accurately track the eventual decision the loser would reap upon themselves if the fight were to continue^11^. These findings lend further support to *theoretical* claims that human psychological systems can forecast the avenue to victory held by specific agonistic behaviours^11^.

There were two methodological advantages to using judges’ decisions as a proxy for perceived RHP^11^. Firstly, to measure perceived RHP, previous research has only used online participants that rate the static stimuli of professional fighters on perceived fighting ability^22,47^. These data are limited to people from contemporary societies wherein exposure to hand-on-hand combat as a method of conflict resolution is likely lower than ancestral populations^14,48^. Using UFC judges’ rulings allowed us to examine whether humans who are exposed to many contests accurately track RHP^11^. Secondly, this approach allowed us to examine a very large sample of 2765 fights, the largest to date in contest competition research (see^11,49^). Given that human contests can end suddenly and unexpectedly, large samples of individual contests are necessary for the statistical power required to detect significant differences in fighting styles on fight outcomes, should they exist.

Despite the extensive experience required to officiate professional mixed martial arts competitions in the UFC, human psychological systems are naturally susceptible to errors and inaccuracies. We found that judges’ decisions did not accurately track grappling skill as an all-round but rather as a submission-typical strategy, potentially because there is a such a strong perception—not reflected in reality—that all grappling behaviours are inextricably tied to submission victories^40^. Given the extent to which grappling vigour is positively associated with submission victories, but negatively associated with knockout victories, human psychological systems might perceive grappling skill as submission-typical strategy as a form of error-management. Lane and Briffa^11^ indeed found that judges overestimate the importance of vigour in contests, especially when compared to skill, suggesting that displays of grappling vigour might overpower perceptions of grappling skill. It should be noted that judges’ rulings for grappling skill as a submission-typical strategy were a very slight inaccuracy given the *p*-value (0.01) was small for a sample size of this magnitude. Nevertheless, we found consistent support for the previously speculated claim that “judges’ ruling should be analogous to the decision the loser would eventually make for themselves if the fight were to continue” ^11^.

While there are a number of strengths to the present work, there are also several limitations. First, the UFC contains weight categories, which can limit the effect of morphological features on fighting performance^23,47^. Future research could examine the few championships in the world that do not have weight restrictions (e.g., Road Fighting Championship). Second, the UFC contains the most elite fighters in the world^23,47^. This might reduce variance in fighting skill and vigour in contests, but it is currently unknown how fighting skill and vigour function in amateur contests. While the UFC is the only organisation to collect fight-specific performance metrics, future research might wish to manually code fighting performance metrics from the contests in other championships. Third, in line with previous research, we measured perceived resource-holding power using judges’ evaluations of fighting performance^11^. UFC judges have extensive experiencing in evaluating contests, and it is currently unknown how naïve raters track fighting skill and vigour. Future research might wish to present online participants with recorded fights, providing participants with a slider that allows them to report their evaluations of fighting performance as the fight progresses.

Our findings stimulate several future lines of enquiry into how human contest competition conform to patterns in the broader animal competition literature. Future research will benefit from empirically examining whether our findings that different indicators of fighting ability in humans reflect unique paths to victory, and hence different forms of RHP, predicts outcomes in nonhuman animal contests. For example, nonhuman animal contests result in either death, which is equivalent to knockout in human contests (^11^; knockouts can result in death:^41,42^) or submission or retreat, considered equivalent to submission in human contests^11^. Consequently, our argument that different measures of RHP are associated with unique paths to victory, that different RHP indicators are distinguished by a unique path to victory, and that third-party observers accurately track fighting vigour and skill along their unique paths to victory represents an exciting new line of enquiry beyond human contest competition into the many domains of animal contest theory.

## Material and methods

We used a publicly available dataset of UFC fights collated from UFCstats.com^50^, which included data from 2765 unique fights (women: 246; men: 2519) held between March 21st, 2010, and 11th July, 2020, from 1392 unique fighters (women: 144; men: 1248). Following Lane and Briffa^11^, we only used data containing fight-specific data (i.e., fighter’s skill and vigour for the specific fight, as opposed to the fighter’s lifetime skill and vigour averaged across all fights). Compared to Lane and Briffa’s^11^ dataset, our sample twice was the size of unique fighters and five times as many unique fights. To our knowledge, this is the largest fight-specific dataset to date across the sports and biological sciences.

Our analyses included: (1) the percent significant strikes landed (termed by the UFC as striking accuracy) as our measure of striking skill (as defined in^11,33^); (2) number of strikes attempted per second (calculated as the total number of strikes attempted divided by fight duration) as our measure of striking vigour (as defined in^11,33^); (3) focal outcome (lose, win) as our measure for the likelihood of victory; (4) KO/TKO (coded as ‘1’) versus submission (coded as ‘2’), to determine whether fighting abilities were KO/TKO or submission-typical manoeuvres; (5) method of resolution (1: decision; 2: KO/TKO; 3: submission), to examine whether third-party observers’ valuations significantly differed from, or were analogous to, a particular avenue to victory; (6) sex (female, male); and (7) fighter ID. We also used fight-specific information for: (8) percent takedowns landed (termed by the UFC as grappling accuracy) as our measure of grappling skill (as defined in^33^); and (9) number of takedowns attempted per second (calculated as the total number of takedowns attempted divided by fight duration) as our measure of grappling vigour (as defined in^33^).

Our focal fighter data comprised 234,643 significant strikes attempted, 101,311 significant strikes landed, 7949 attempted takedowns, 2998 landed takedowns, which ensures statistical stability to the larger variables to which they belong (i.e., skill and vigour) and emphasises the advantages of using UFC data in contest competition research. The UFC (a billion-dollar organisation) is the only organisation to collect fight-specific performance data, and across a large sample of contests, which would ordinarily be extremely expensive to collect through other sampling methods (e.g., simple random sampling).

Lane and Briffa^11^ argued that a fighter’s skill and vigour are likely dependent on their opponent’s fighting behaviour; to address this, they used from one ‘focal’ individual per fight. In line with Lane and Briffa^11^ then, we used data from one ‘focal’ individual per fight with ‘fight’ as the level of replication. Focal fighters were randomly assigned by generating random numbers for the red and blue fighters to avoid potential confounding effects of fighter colour on fighting success^11^. We used Excel’s RANDBETWEEN function to randomly generate numbers ‘1’ or ‘2’ for each of the 2765 fights, with red fighters being selected as the focal fighter for ‘1’ and blue fighters selected as the focal fighter for ‘2’.

We used the R code provided by Lane and Briffa^11^ to conduct identical generalized linear mixed effects models (GLMMs) via backwards elimination (to remove non-significant terms) for striking skill and vigour, which was then adapted for grappling skill and vigour, with outcome (win vs. lose) as our outcome variable. These GLMMs also included sex and the method of resolution, with the latter entered as a factor to examine Hypotheses 5 and 6. To examine Hypotheses 3 and 4, we ran similar GLMMs but with KO/TKO versus submission as a predictor variable on the likelihood on victory, with a significant positive interaction between fighting skill or vigour and KO/TKO versus submission indicating that the fighting manoeuvre was a submission-typical strategy, a significant negative interaction indicating a KO/TKO-typical strategy, and no significant interaction indicating an all-round strategy to victory (an interpretation predicated on the initial finding that the fighting manoeuvre is associated with the likelihood of victory; as we have demonstrated below, striking and grappling skill and vigour are all associated with the likelihood of victory). There was no strong pattern in the residuals nor any indication of overdispersion in our results. In line with Lane and Briffa^11^, random intercepts were included to account for the ID of both fighters (red and blue) per fight. Owing to their different scales, striking and grappling skill and vigour were *Z*-standardised prior to analysis. Our R code and data is available on the Open Science Framework (https://osf.io/be85y/).

## Data Availability

Our R code and data is publicly available on the Open Science Framework (https://osf.io/be85y/).
